# Rational Biological Interface Engineering: Amyloidal Supramolecular Microstructure-Inspired Hydrogel

**DOI:** 10.3389/fbioe.2021.718883

**Published:** 2021-07-19

**Authors:** Qize Xuan, Yibing Wang, Chao Chen, Ping Wang

**Affiliations:** ^1^State Key Laboratory of Bioreactor Engineering, School of Biotechnology, East China University of Science and Technology, Shanghai, China; ^2^Department of Bioproducts and Biosystems Engineering, University of Minnesota, Saint Paul, MN, United States

**Keywords:** amyloid fibrils, hydrogel, self-assembly, tissue engineering, drug delivery

## Abstract

Amyloidal proteins, which are prone to form fibrillar and ordered aggregates *in vivo* and *in vitro*, underlie the mechanism for neurodegenerative disorders and also play essential functions in the process of life. Amyloid fibrils typically adopt a distinctive β-sheet structure, which renders them with inherent extracellular matrix (ECM)-mimicking properties, such as powerful mechanical strength, promising adhesion, and antibacterial activity. Additionally, amyloidal proteins are a category of programmable self-assembled macromolecules, and their assembly and consequent nanostructure can be manipulated rationally. The above advantages motivate researchers to investigate the potential of amyloidal proteins as a novel type of hydrogel material. Currently, the amyloid-inspired hydrogel has become an emerging area and has been widely applied in a variety of biomedical fields, such as tissue repair, cell scaffolds, and drug delivery. In this review, we focus on the discussion of molecular mechanisms underlying the hydrogenation of amyloidal proteins, and introduce the advances achieved in biomedical applications of amyloid-inspired hydrogels.

## Introduction

Amyloidal proteins possess different primary sequences and physiological functions but can assemble into similar fibrillar aggregates. Folded by non-covalent interactions among backbones and side chains, these amyloidal proteins are typically stacked into parallel or antiparallel in-register intermolecular β-sheet or rare α-helix architectures (for example, PSMα3) to form fibrous protein aggregates called amyloid fibrils (Sawaya et al., 2007; Kajava et al., 2010; Wang et al., 2012; Yu et al., 2020). Initially, amyloid fibrils are one of the main compositions identified from the pathological regions of protein misfolding diseases, such as Alzheimer’s diseases (AD), type II diabetes (T2D), and Parkinson’s disease (PD) (Knowles et al., 2014). Interestingly, amyloid fibrils are also proved to be beneficial for vital activities of living afterward (Chaturvedi et al., 2016), and they have already been widely utilized to accomplish colonization and/or survival by microbes and other lower organisms (Deshmukh et al., 2018). For example, Curli, the first observed and studied functional amyloid in *Enterobacteriaceae*, is proved to be essential for initial adhesion to biotic and abiotic surfaces and is also associated with environmental resistance (Erskine et al., 2018).

The vital physiological roles of amyloid fibrils within living cells depend on their unique assembled structure in the extreme. Essentially, amyloid fibrils adopt a cross-β structure where stacked β-strands comprise the axis of fibrils and β-sheets are strictly vertical to the axis. Such cross-β structures are firmly stabilized by numerous types of non-covalent interactions, such as hydrophobic interaction, π–π stacking, and electrostatic interaction, which contribute to the formation of protofibrils and subsequent mature fibrils with a length of several micrometers through phase transformation procedures (Schleeger et al., 2013). These cross-β structures endow amyloid fibrils superior mechanical strength. Meanwhile, these non-covalent interactions within amyloid fibrils endowed them with controllable assembly characteristics. Besides, the highly repetitive arrangement of multiple residues exposed peripherally and unnecessary for β-sheet formation on the surface provides abundant binding regions, contributing mostly to the binding capacity and further entanglement of amyloid fibrils (Das et al., 2018). These tangled fibrils further form three-dimension fibrils networks, which can capture amounts of water, and this is owed to the subtle balance of hydrophobicity and hydrophilicity in sequences of amyloidal proteins in the hierarchical bottom-up self-assembly process (Zhang et al., 2014). These superior properties derived from the organized structure of amyloid fibrils, such as high mechanical strength, controllable assembly, multiple bonding, and water-retaining ability encourage researchers to think about their applications in biomaterials. For example, Cheetham et al. (2013) utilized Tau fibrils and CPT (an anticancer drug) to co-assemble into stable and uniform nanostructures, which enhanced drug loading capacity and in vitro killing activity against multiple cancer cells efficiently.

Hydrogel, as a non-invasive form in biomedicine, is attracting more and more attention, owing to a tunable physicochemical property and an ECM-mimicking characteristic (Fan and Gong, 2020). Among them, supramolecular injectable hydrogels are widely studied in the fields of drug delivery, tissue repair, and 3D cell culture (Li et al., 2019). Amyloid fibrils are believed to be significant gelators for fabricating supramolecular hydrogels (Das et al., 2018). (1) The robust mechanics of amyloid fibrils confer hydrogel the tunable mechanical property by changing the number of β-sheets with altering physicochemical factors of the assembly process, which provides a smart platform for directing stem cell differentiation. (2) Highly ordered residues of amyloid fibrils exposed on the surface and resembled extracellular matrix (ECM) endow hydrogel a perfect sticking property, making it turn into a benign cell scaffold, especially for cell adhesion and spreading. (3) Non-covalent interactions supporting fibrils formation provide thixotropy and injectability for hydrogel, which are essential for clinical utilization, particularly in drug delivery. (4) The unique structures of amyloid fibrils entitle hydrogel to have a capacity to kill bacteria just like antimicrobial peptides (AMPs), making it useful in tissue repairs, for example, in chronic wound highly susceptible to microbial infections. This review outlines recent efforts that have been directed to the amyloid-inspired hydrogel applied in the above fields, such as stem cell differentiation, cell adhesion and spreading, drug delivery platform, and antibacterial agent. The underlying structural basis and unique nature in corresponding applications are also elucidated in details (Scheme 1).

**FIGURE 1:**
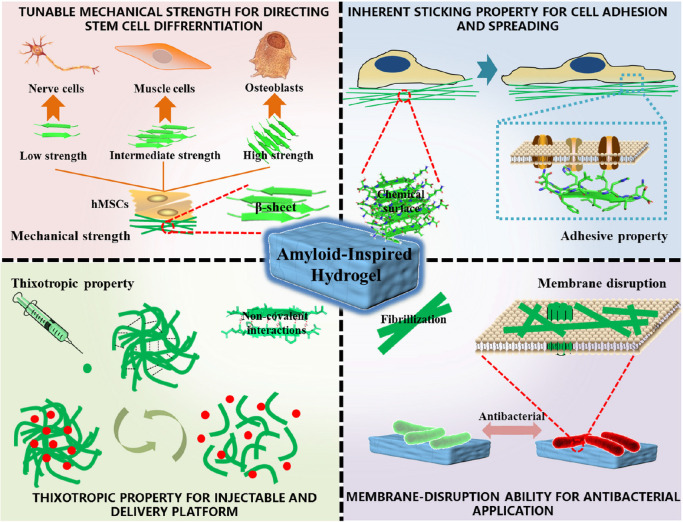
Structural basis, excellent properties, and corresponding biomedical applications of amyloidal supramolecular microstructure-inspired hydrogel.

### Tunable Mechanical Strength of Amyloid-Inspired Hydrogel for Directing Stem Cell Differentiation

The entanglement of amyloid fibrils forms a 3D network with retained amounts of water, which makes it easier to fabricate hydrogels (Das et al., 2018). Besides, such unique and organized β-sheet structures in amyloid fibrils bring superior mechanical properties (3.3 ± 0.4 GPa), exhibiting Young’s modulus similar to that of silk (1–10 GPa) (Smith et al., 2006; Knowles et al., 2007). From the viewpoint of molecular interaction, the rigidity of amyloid fibrils mainly comes from the regular intermolecular hydrogen bond network in the β-sheet cores, and non-covalent interactions between the side chains of residues (π–π stacking, hydrophobic and electrostatic interactions) will further stabilize the fibrous structure and improve their Young’s modulus (Schleeger et al., 2013). The rigidity of amyloid fibrils provides an essential basis for the mechanical strength of amyloid-inspired hydrogel, and these are substantially ascribed to β-sheet structures in amyloid fibrils. In addition, inter-crosslinking of amyloid fibrils further facilitates the mechanical intensity of hydrogel, in which the protein concentration dominated the β-sheet contents and crosslinking conditions (Ruggeri et al., 2015). Rigidity-directed and crosslinking-controlled strategies for the mechanical regulation of amyloid-inspired hydrogel are both put forward (Wang et al., 2021). In the rigidity-directed strategy, residue substitution is a practical method. Fibrils with high mechanical strength usually contain a dense hydrogen-bonding network in the β-sheet core, and thus, some residues or motifs with β-sheet propensity are more likely to be selected in this core area, such as isoleucine (Ile), phenylalanine (Phe), and tryptophan (Trp), or a specialized motif (NNQQNY) (Zhang et al., 2014). In addition, the types of residues in the β-sheet core can affect an intermolecular non-covalent interaction, which is mainly caused by the hydrophobicity, polarity, charge, and steric hindrance of the side chains of amino acids (Wang et al., 2021). Owing to the programmability of original amyloid fragments, the rational regulation of the mechanical strength of fibrils can be achieved. In the crosslinking-controlled strategy, by changing the concentration of proteins and further affecting the number of β-sheet and cross-linking degree of amyloid fibrils, the mechanical strength of amyloid-inspired hydrogel can also be rationally tuned (Ruggeri et al., 2015; De Leon Rodriguez et al., 2016).

The different mechanical strengths of hydrogel can affect the differentiation of stem cells. Stem cells are referred to as a class of primitive undifferentiated cells with multidirectional differentiation potential and self-replication ability to generate the tissues and organs of mammals. Among them, human mesenchymal stem cells (hMSCs), originally found in marrow, are pluripotent stem cells, which still keep common traits of all stem cells, such as multidirectional differentiation potential, self-replication capacity, and regulation ability in hematopoietic and immune aspects. Therefore, hMSCs have become promising candidates for cell therapy, and nearly 1,000 related clinical trials have been executed or completed (Tsai et al., 2020). Differentiation directions of hMSCs are not only affected by intracellular DNA reprogramming but also influenced by the *in vivo* microenvironment of cell growth. Three key interactions in a niche (*in vivo* microenvironment), namely, growth factors, cell-cell contacts, and cell-matrix adhesions, constituted the significant factors for hMSCs differentiation (Discher et al., 2009). Among these factors, the mechanical property of the matrix in cell-matrix adhesions is proved an essential factor affecting the differentiation of hMSCs (Wen et al., 2014). For example, a matrix with low mechanical strength (0.1–1 kPa) can induce the differentiation of hMSCs into nerve cells, and a matrix with intermediate strength (1–25 kPa) can entice hMSCs to differentiate into the direction of the muscle cells, and the higher mechanical strength (25–40 kPa) of the matrix may lure hMSCs into osteoblast. This is because the non-muscle myosin II subtypes (NMM type IIA, B, and C) on the surface of hMSCs can transport the mechanical information of ground substance into cells *via* the formation of local adhesion or local adhesion complexes between cells and the matrix (Engler et al., 2006).

Amyloid-inspired hydrogels show excellent performance in the respects of adjustable mechanical strength, which open up novel avenues to realize the directional differentiation of hMSCs according to the preset target. Das et al. (2016) generated a series of building blocks based on the amyloidogenic region of α-synuclein (residues 74–78) *via* a residue substitution method to construct the hydrogel. Among them, hMSCs incubated on the hydrogel matrix fabricated by Fmoc-VYAVA (∼0.1 kPa) and Fmoc-VHAVA (∼1 kPa) exhibited significantly neuron-like morphological characteristics, compared with other hydrogels and glass matrix (60–80 GPa). From the viewpoint of cell morphology, cells cultured on these two hydrogels had less roundness but more elongated morphology compared with those in control groups, and Fmoc-VYAVA-based hydrogel showed extended bipolar morphology from an earlier culture, with further extension and branching over time. To further identify the differentiation lineage of hMSCs, they evaluated the expression of genes related to neuronal differentiation after being cultured on Fmoc-VYAVA-based hydrogel for 5 days using glass as a control. High expression levels of tubulin β3 (TUBB3, neural markers) and low expression levels of glial fibrillary acidic protein (GFAP, astrocyte markers) demonstrated that Fmoc-VYAVA-based hydrogel promoted the differentiation of hMSCs into neuronal lineages, as shown in Figure 1A (Das et al., 2016). In addition to the residue substitution strategy, an increase in concentration will significantly increase the crosslinking degree of fibrils rich in β-sheet, thus improving the mechanical strength of amyloidal hydrogel. Similarly, Jacob et al. (2015) constructed a series of amyloid-inspired hydrogels with different concentrations based on Fmoc-VIV, which is derived from amyloidogenic fragments of amyloid β (Aβ, residues 40–42). The results showed an obvious linear relationship between the building block (Fmoc-VIV) concentration and the mechanical properties of the corresponding hydrogel (G’–C^1^.^7^, C refers to concentration: mg/ml). The low mechanical strength (∼0.02 kPa) of the Fmoc-VIV-based hydrogel (6 mg/ml) can promote hMSCs toward neuronal differentiation, displaying significant bipolarization in cell morphology, accompanied by the expression of β-III–tubulin, compared with the control group, and the expression of specific markers associated with neural differentiation volume was also significantly enhanced (Jacob et al., 2015).

**FIGURE 1:**
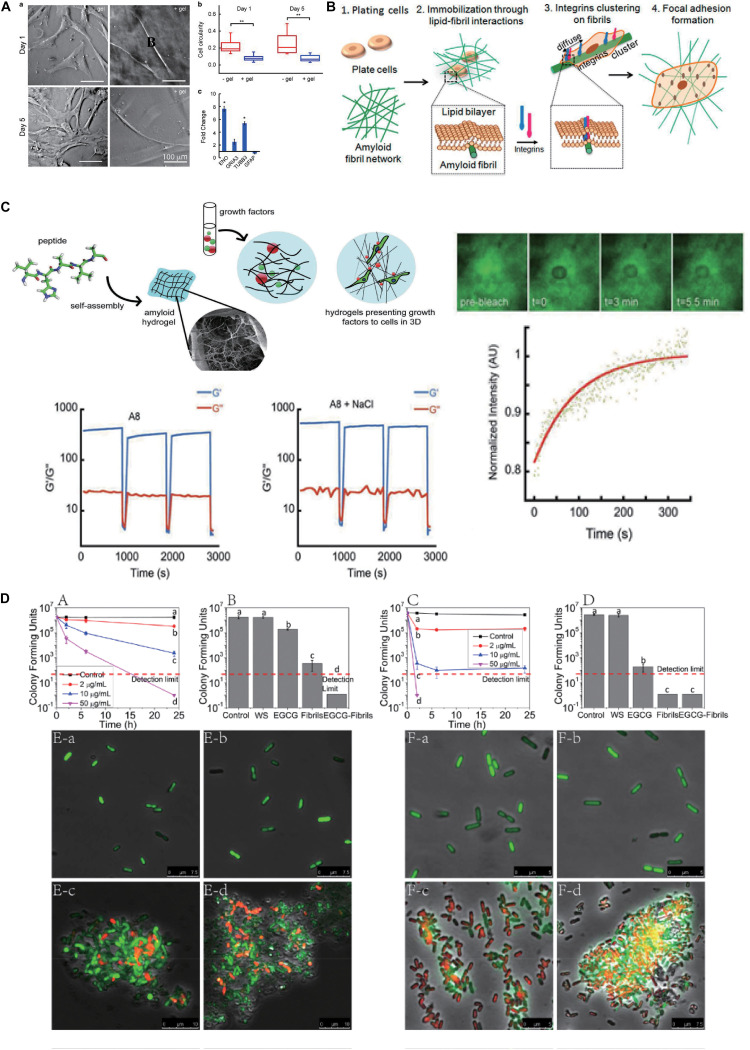
Biomedical applications of amyloidal-inspired hydrogel in previous reports. **(A)** Differentiation of mesenchymal stem cells with amyloid hydrogels. Morphology, cell circularity, and expression of genes related to neuronal differentiation characterization of hMSCs incubated on Fmoc-VYAVA-based hydrogel and glass matrix. Student’s *t*-test was performed to evaluate the difference between two groups. Statistical significance was defined as *p < 0.05, **p < 0.01. **(B)** Schematic of the mechanism of cell spreading on amyloid fibrils. **(C)** Schematic of peptide monomer self-assembling to form amyloid hydrogels with distinct pore structures, which can encapsulate growth factors for sustained release for stem cells differentiation. Step-strain oscillatory rheology proved that the Fmoc-VHAVA-based hydrogel network was disrupted under high strain (100%) and then recovered under low strain (0.05%) for three cycles. Adapted with permission from Das et al. (2017) (Copyright© 2017 Wiley-VCH Verlag GmbH & Co. KGaA, Weinheim). **(D)** Antibacterial activity by the agglomeration effect of the polyphenol-binding lysozyme amyloid fibrils (hydrogels). Colony-forming unit counting and confocal laser scanning microscopy (CLSM) results reveal that polyphenol-binding lysozyme amyloid fibrils (hydrogels) show superior antibacterial capacity on both *E. coli* and *Listeria monocytogenes*. Adapted with permission from Hu et al. (2018) (Copyright© 2018, American Chemical Society). Significant differences among different treatments on reducing CFUs determined with one-way ANOVA followed by Tukey’s test, *p* ≤ 0.05, are indicated by different lowercase letters in A–D.

### Inherent Sticking Property of Amyloid-Inspired Hydrogel for Cell Adhesion and Spreading

Amyloid fibrils are a kind of biocompatible material with inherent cellular sticking ability, which is derived from its unique surface containing a highly ordered β-sheet structure, enabling it to bind to different biological macromolecules, such as polysaccharides, DNA, and proteins (Das et al., 2018). The typical cross-β structure not only gives amyloid fibrils great mechanical strength, by which residues buried in the β-sheet core within the fibrils form dense intermolecular interactions, it also provides unique surface characteristics, by which the side chains of residues unnecessary for β-sheet formation spread out the main body of fibrils and mold a chemical surface rich in charged and hydrophobic side chains and highly ordered nanotopography (Reynolds et al., 2013). These two parts offer the structural basis for the cellular adhesion properties of amyloid fibrils, and this cross-β structural property of amyloid fibrils had been widely utilized by many lower organisms (Reynolds et al., 2015). Such as, cement protein 19k derived from barnacle self-assembled into enriched β-sheet structures, exhibiting an adhesive ability stronger than that of the unordered state of the same protein (Liang et al., 2018). The Curli protein secreted by *Escherichia coli* is essential for the initial adhesion of *E. coli* on biological and non-biological surfaces, and is the decisive factor for the formation of *E. coli* biofilm (Erskine et al., 2018). It can also assemble into cross-β amyloid fibrils to further enhance their adhesive ability, which may be due to the increase in the interfacial contact area and cohesive strength caused by cross-β amyloid fibrils (Zhong et al., 2014).

Cellular adhesion materials have profound significance in bone tissue repair, wound dressings and stem cell implantation, etc. Adhesion of cells *in vivo* is dependent on the interaction between integrin on the surface of the cell and ECM -derived proteins (such as collagen, laminin, and fibronectin), and this is because ECM-derived proteins generally contain integrin-recognition motif. Although amyloid fibrils do not contain integrin-binding motif, the unique surface properties enable them to function like ECM (Das et al., 2018). Many studies have shown that the cell adhesion ability of amyloid fibrils is not picky and independent of integrin motif, and various mammalian cells can adhere and stretch by the abduction of their sharing nanotopology and surface chemistry characteristics (Jacob et al., 2016). Hence, different amyloids commonly exhibit adhesion property; what is more, most of which precede collagen-based materials in cell adhesion and spread. The sticking property of amyloid fibrils rich in cross β-sheet structure endowed amyloid-inspired hydrogel composed of interior interlaced and entwined amyloid fibrils with great adhesive performance, which displayed the immense potential of the amyloid-inspired hydrogel as cell scaffold or adhesives.

The adhesive capacity of amyloid fibrils depends on cross β-sheet structure, and this fact is universal in various amyloidal peptides. Jacob et al. (2016) tested approximately 20 kinds of amyloid fibrils, such as helodermin, kassinin, α-synuclein, and β-endorphin. The results (Figure 1B) showed that all of them adopted the form of amyloid fibrils that had greater excellent cell adhesion ability compared with their soluble states, and even some types of amyloid fibrils promoted the high expression of the β1 integrin. Interestingly, they found that bovine serum albumin (BSA), which is often used to repel cell adhesion, showed superior adhesion of cells when transformed into amyloid fibrils. Their results proved that cell adhesion ability was an inherent property of amyloid fibrils (Jacob et al., 2016). The nanotopology of amyloid fibrils also contributes to promoting cell adhesion in the above-mentioned structural basis. Reynolds et al. (2013) masked the TTR1 assembly substrate using a non-specific coating layer of DGpp. Such an approach maintains the topology of amyloid fibrils on the substrate but shields out the interactions encoded by amyloid proteins (Reynolds et al., 2013). They found that there were obvious differences in cell adhesion on three amyloid fibrils without DGpp deposition (TTR1 = 1.1 ± 0.073, TTR1-CRGD = 1.9 ± 0.18, and TTR1-RGD = 0.99 ± 0.13, a ratio of attached cells to unmodified mica sheet). However, there were no significant differences in cell adhesion among the three groups (TTR1-DGpp = 1.48 ± 0.25, TTR1-RGD-DGpp = 1.47 ± 0.25, and TTR1-cRGD-DGpp = 1.28 ± 0.15, a ratio of attached cells to DGpp-modified mica sheet) when DGpp was deposited, indicating that the nanotopology of the amyloid fibril surface contributed most to the cell adhesion of amyloid fibrils (Reynolds et al., 2015).

### Thixotropic Amyloid-Inspired Hydrogel for Delivery Platform

The amyloid sequences provide abundant non-covalent interactions for hydrogel formation, in which the fibril structure forms primarily *via* self-assembly of building blocks followed by hydrogel generation through amyloid fibrils entanglement. Although the cross-β structure of amyloidal fibrils are stable, the entanglement of amyloidal fibrils is totally dependent on the non-covalent interactions of side residues, and they are vulnerable to environmental conditions and showed dynamic transformation along with changes in external circumstances (Yan et al., 2010). For example, the hydrogel solution transformation can be well regulated *via* affecting non-covalent interactions in the formation of amyloid-inspired hydrogel by shear force. When the shear force is applied, the three-dimensional structure composed of fiber tangles (hydrogel state) will be fractured into domains because of their fragility derived from non-covalent interactions, and then with the further increase in shear force, the stable interactions inside the fibrils may also be damaged, resulting in truncation or partial depolymerization of the fibril structure. The time required for self-healing of hydrogel depends on the damage degree of its assembly structure. Owing to the strong mechanical strength of amyloidal fibrils themselves, most of them could survive and serve as a seed to further accelerate the subsequent assembly. Hence, the self-healing time of amyloid-inspired hydrogel is relatively short (Yan et al., 2010).

This shear force-induced state change in the hydrogel is termed thixotropy. The thixotropy of hydrogel is defined based on liquid viscosity, which provides the physical basis for their injectability, and the latter is particularly emphasized in the biomedical application of protein-based hydrogel. Thixotropy refers to the process in which the viscosity of hydrogel decreases and the state of hydrogel changes from solid to liquid after exerting a certain shear force, and then the state of hydrogel changes from liquid to solid and the viscosity increases again when the shear force is decreased or eliminated. Studies have shown that the microstructure of peptide-based hydrogel will change greatly, exhibiting a decrease in the nanofiber amount and an increase in irregular curl structure, with the increase in shear force. Hydrogel recovery kinetics is closely related to the shear rate, shear duration, and hydrogel hardness before shear. The thixotropy of peptide-based hydrogel, especially those formed by short peptides, originated from non-covalent interactions as the main driving force in their self-assembly process, containing hydrogen bonding, π–π stacking, electrostatic effects, and hydrophobic interactions, etc (Yadav et al., 2020). These effects tend to be weak and vulnerable to shear stress and show dynamic changes accompanied by variation in shear force (Zanna and Tomasini, 2017). Shear force is input in this hydrogel system as external energy, making the system no longer thermodynamically isolated, and distance-dependent intermolecular interactions deviate from the chemical equilibrium state, which ultimately leads to a state comparable to infinite dilution with no likelihood of rebinding. However, when the shear stress terminates, the interactions will reform, and the system can go back to equilibrium progressively through molecular motion (Evans and Ritchie, 1997).

Protein-based hydrogels have momentous practical values in tissue engineering, regenerative medicine, and drug delivery (Acar et al., 2017; Yang et al., 2019). In these fields, hydrogels generally need to be injected into damaged areas of the body by carrying cells or drugs. Because of its excellent thixotropy, amyloid-inspired hydrogel is easy to inject in liquid at the target site and then recovers to solid hydrogel, which is conducive to its wide application in biomedicine. In this way, amyloid-based hydrogels can act as a repository for the local release of therapeutic molecules or incubation of stem cells, providing attractive alternatives to present delivery platforms. Compared with other platforms, amyloid-inspired hydrogels have advantages in controlled fabrication, retaining site-specific conformation, supporting surrounding tissue, and reducing the invasive nature of an implant. Thixotropic amyloid-based hydrogel is a promising delivery platform for drug or stem cells with great potentials in controlled release and cell implantation. Yang et al. (2019) prepared an amyloid-based hydrogel with tightly coiled amyloid fibers successfully, utilizing fragments derived from hen egg white lysozyme (HEWL) regulated by magnesium ions. In the rheological analysis of a step strain-time variation, the hydrogel retained solid phase under 0.1% strain. When the strain was increased to 40 and 800%, the hydrogel disrupted and transformed into the solution phase, and recovered after the strain was adjusted to 0.1%, which showed great thixotropy and provided a foundation for its injectability and drug delivery. Using DOX as a model drug, this amyloid hydrogel was able to be injected and achieved a 100% drug loading efficiency *via* a simple blending method, and 50% drug release within 12 h (Yang et al., 2019). Besides functioning as drug carriers, amyloid hydrogels have also been utilized for carriers of growth factors to promote the differentiation of stem cells. Das et al. (2017) prepared Fmoc-VHAVA-based hydrogel after modification of the NAC domain of α-synuclein. Step-strain oscillatory rheology proved that this hydrogel network was disrupted under high strain (100%) and then recovered under low strain (0.05%) for three cycles. Then, they measured the slow-release properties of the hydrogel using FITC-BSA as the model protein and found that the hydrogel could release FITC-BSA continuously for up to 30 days. Then, they performed 3D culture of hMSCs in the Fmoc-VHAVA-based hydrogel loading growth factors fibroblast growth factor 8 and sonic hedgehog, enabling the targeted differentiation of hMSCs into nerve cells, as shown in Figure 1C (Das et al., 2017). Likewise, stem cells can also be readily delivered to the desired location through invasive injection of the amyloid-based hydrogel. Das et al. (2016) designed and prepared Fmoc-VYAVA-based hydrogels carrying GFP-hMSCs, and the oscillatory rheology with cyclic 100% and 0.5% strain at a constant frequency of 1 Hz shows that this hydrogel is thixotropic. Then, it was injected into the brain of a Parkinsonian mouse and formed solid hydrogel in the desired location. The results showed that the number of GFP-positive cells in the striatum region (108 ± 12 vs. 34 ± 2 cell numbers per section) and the substantia nigra (SN) region (82 ± 4 vs. 20 ± 3 cell numbers per section) were significantly higher than those in the control group (cells were distributed in PBS and injected), and that the distribution area of cells was also significantly improved (∼6 × 10^4^ m^2^ vs. ∼2 × 10^4^ m^2^). In addition, high expression of β-III tubulin, a neuro-specific marker, was observed in the amyloid-based hydrogel injection group (Das et al., 2016).

### Unique Structural Properties of Amyloid-Inspired Hydrogel for Antibacterial Application

The implementation of the antibacterial activity of an amyloid depends on its specific sequence, self-aggregation property, and the typical cross β-sheet structure. The amino acid sequence of amyloids affects their self-aggregation property and dominates the formation of the β-sheet structure, thus directing the process of amyloid fibrillation (Zhang et al., 2014). Recent studies have reported that amyloid showed excellent antibacterial activity, and its resistance to several common microorganisms was even better than typical antimicrobial peptides in some cases, such as LL-37, which is a natural immune peptide with an amphiphilic α-helix structure and good antimicrobial ability, secreted by various types of cells, such as macrophages and neutrophils (Last and Miranker, 2013). Many antimicrobial peptides self-assemble to form assemblies rich in β-sheet structure to achieve their antibacterial activity. Thus, it can be seen that the β-sheet structure is vital for the achievement of the antimicrobial activity of amyloid. Historically, the antibacterial activity of amyloid is probably derived from a protective mechanism of the human body against microbial infection, reported by many studies. For example, in mouse and nematode models, Aβ can kill *Salmonella typhimurium* and *Candida albicans* to protect cells against infection, and a study also found that the assemblies in different aggregation stages perform various functions during the antibacterial process. Aβ oligomers mainly targeted polysaccharides on the cell wall of microbes through its heparin-binding domain, while Aβ protofibrils inhibited the binding of pathogenic microorganisms to host cells, and mature fibrils mediated the aggregation, capture, and killing of pathogenic microorganisms (Kumar et al., 2016).

The resistance to microorganisms of amyloid generally occurs *via* membrane-disrupting mechanisms, mainly including detergent-like membrane disruption and membrane thinning. The amphipathicity of amyloid is essential for detergent-like membrane disruption, in which residues with positive charge can conduce to interfacial adoption on anionic membranes of microorganism, and the lipid membranes, in turn, accelerate fibrillization of amyloid. Such intensive distribution and interaction of the amyloid fibrils in the membrane will dislodge wrapped lipids from the membrane, and thus, decrease the interfacial tension of the membrane and finally result in membrane fragmentation. Besides, membrane-bound amyloid fibrils also can work *via* a membrane-thinning mechanism. These large membrane-anchored amyloid fibrils can heavily contort the membrane shape of microorganisms, or even tune their orientations, i.e., orienting partial insertion of the hydrophobic region into the hydrophobic core of the membrane, ultimately leading to membrane thinning and subsequent membrane disruption (Zhang et al., 2014).

Amyloid fibrils show excellent intrinsic antibacterial activity, which is of great potential to construct amyloid-based hydrogels for antibacterial applications. Lysozyme is well known to kill Gram-positive bacteria *via* hydrolysis of 1,4-β-linkages between N-acetylmuramic acid and N-acetyl-D-glucosamine residues in peptidoglycan in the cell wall, while it nearly has no resistance to Gram-negative bacteria. To further prove that the amyloid fibril form of lysozyme brings the ability to combat Gram-negative bacteria, Hu et al. (2018) used a lysozyme monomer, lysozyme amyloid fibrils, and short fibrils prepared through homogenization of mature lysozyme amyloid fibrils for testing, and the data revealed that lysozyme amyloid fibrils and homogenized short fibrils displayed greater antibacterial activity compared native lysozyme protein monomers, suggesting that hydrophobic, cationic, and conformational properties of the amyloid fibrils dominated the observed antibacterial activity. Next, they fabricated supramolecular hydrogel composed of lysozyme amyloid fibrils and epigallocatechin gallate (EGCG) through collaborative assembly, and the hydrogel showed outstanding antibacterial ability, which decreased the number of *E. coli* (Gram negative) and *Listeria monocytogenes* (Gram positive) by more than four orders of magnitude, corresponding to 99.99% antimicrobial efficiency, for 24 and 2 h, respectively. These were attributed to the combined effects of lysozyme fibrils (50 μg/ml) and EGCG (4.06 μg/ml). In addition, lysozyme amyloid fibrils (50 μg/ml) alone also achieved a more significant antibacterial effect (both *E. coli* and *L. monocytogenes*) in this hydrogel system, which could entrap bacteria and destroy the integrity of cell membrane, so as to achieve the powerful antibacterial activity, as shown in Figure 1D (Hu et al., 2018). Lysozyme amyloid fibrils-based hydrogels have been widely used as scaffolds in tissue engineering and bone repair fields without significant cytotoxicity (Reynolds et al., 2014). Therefore, amyloid fibril-based hydrogel has a great potential for application in the biomedical field, for example, wound dressings, due to their good antimicrobial ability and biocompatibility.

## Conclusion and Perspectives

Amyloid fibrils are widespread in nature and are relying on their function and properties, which enable them to be developed in inter-disciplines ranging from physics and chemistry to biomedicine and materials. As a kind of excellent building block, amyloid has been widely utilized for hydrogel fabrication, and these amyloid-based hydrogels have been applied in many spheres, such as stem cell differentiation, cell adhesion and scaffold, delivery platform, and antibacterial agents. All these applications were based on various superiorities of amyloid, and all of which were attributed to their unique cross β-sheet structure. This review emphasized the structure-function-application relationships and outlined recent efforts in the development of amyloid-based hydrogel.

Although amyloid-based hydrogels have shown a great potential in biomedicine, related studies still remain rare. Some key concerns remain to be solved. To date, few amyloid-derived peptides selected for hydrogel formation overstepped the derivation of an already mature system, most of which are derived from known biological architecture instead of integrated design freedom. This is because it is still a challenge to predict and acquire desired hydrogel materials directed by completely free design, especially external stimulus-responsive hydrogel fabrication. In addition, just like other peptide-based hydrogels, amyloid-based hydrogels are still limited by their fragility derived from their protein nature in some scenarios, and more efforts should be made to further improve their mechanical strength to expand the scope of their application. It is also necessary to accelerate the clinical transformation of the amyloid-based hydrogel. The long-term stability, long-term biosafety, and pharmacokinetic evaluation of amyloid hydrogels need to be further systematically studied. Among, long-term biosafety is of particular concern in biomedical applications, especially these hydrogels made by neurodegenerative diseases associated amyloids because these diseases develop rather slowly.

## Author Contributions

QX researched the literature and wrote the review. All authors revised and polished the review.

## Conflict of Interest

The authors declare that the research was conducted in the absence of any commercial or financial relationships that could be construed as a potential conflict of interest.
